# Interventions Initiated Before and After Pregnancy for Women who Experience Severe Nausea and Vomiting of Pregnancy: A Scoping Review

**DOI:** 10.1007/s10995-025-04113-7

**Published:** 2025-05-27

**Authors:** Zeinab El-Dirani, Kurdo Araz, Ola Bazzi, Noreen O’Leary, Grainne Kent, Melanie Nana, Catherine Williamson, Joan Devin, Eileen O’Brien, Angela C. Flynn

**Affiliations:** 1 School of Population Health, Royal College of Surgeons in Ireland, Beaux Lane House, Lower Mercer Street D02 DH60 Ireland, Dublin, Ireland; 2 School of Medicine, Royal College of Surgeons in Ireland, Dublin, Ireland; 3 American University of Beirut, Beirut, Lebanon; 4 Department of Women & Children’s Health, School of Life Course & Population Sciences, King’s College London, London, SE1 7EH UK; 5 Institute of Reproductive and Developmental Biology, Imperial College London, London, W12 0NN UK; 6 Irish Medicines in Pregnancy Service, The Rotunda Hospital, Dublin 1, Ireland; 7 School of Pharmacy and Biomolecular Sciences, Royal College of Surgeons in Ireland, Dublin, Ireland; 8 School of Biological and Health Science, Technological University Dublin, Dublin, Ireland

**Keywords:** Nausea and vomiting of pregnancy, Hyperemesis gravidarum, Postpartum, Preconception

## Abstract

**Introduction:**

Nausea and vomiting of pregnancy (NVP) affects up to 90% of women, while hyperemesis gravidarum (HG), a severe form of NVP, impacts quality of life, and ability to eat and drink normally, with reported recurrence rates up to 89% in subsequent pregnancies. Severe NVP has a profound impact on maternal physical and mental health, impairing daily functioning and quality of life, and is associated with anxiety and depression.

**Aims:**

To conduct a scoping review to identify and characterise interventions initiated before and after pregnancy that aim to mitigate the impact and consequences of severe NVP on maternal health.

**Methods:**

A comprehensive search was conducted across seven electronic databases and included grey literature without restrictions on language or date. Eligible studies were identified according to a prespecified criteria. All references were screened independently by two reviewers.

**Findings:**

Three studies were included; two utilised pre-emptive counselling and antiemetic treatment beginning before pregnancy or in early pregnancy/upon recognition of pregnancy, while one focused on post-pregnancy writing therapy. Both pre-emptive interventions reported a reduction in NVP symptom severity and a lower recurrence rate of HG, while writing therapy was beneficial in aiding recovery from severe NVP and allowed women an opportunity to externalise and process the experience.

**Discussion:**

This study revealed a paucity of interventions initiated before and after pregnancy for women with severe NVP. The included studies showed some benefits of pre-emptive treatment and writing therapy.

**Conclusion:**

Tailored pre-pregnancy and postpartum interventions for women with previous severe NVP are urgently needed to address the physical and mental health burden of the condition.

## Significance

Despite the profound physical and mental health effects of HG and a reported recurrence rate of up to 89% in subsequent pregnancies, there is limited knowledge of effective interventions initiated before and after pregnancy. This study highlights the scarcity of such interventions, identifying two pre-emptive pharmacological and counselling interventions that reduced NVP symptom severity and lowered HG recurrence and one post-pregnancy writing therapy intervention that aided in recovery and provided an opportunity for women to externalise and process their experience.

## Introduction

Nausea and vomiting of pregnancy (NVP) affects up to 90% of women (Bustos et al., 2017; Gadsby et al., 2019). NVP typically begins between 4 and 7 weeks of gestation, peaking in week 9 and resolves by 20 weeks in the majority of women (Fejzo et al., 2019). Hyperemesis gravidarum (HG) is a severe form of NVP, impacting quality of life, ability to eat and drink normally, and affects between 0.3 and 3.6% of women (Einarson et al., 2013; Fejzo et al., 2019) with reported recurrence rates in subsequent pregnancies from 15 to 81% (Dean et al., 2020). Hypersensitivity to the hormone growth differentiation factor-15 (GDF-15) has been shown to be implicated in the pathogenesis of severe NVP (Fejzo et al., 2024).

Severe NVP has a profound effect on maternal physical and mental health. Severe sickness impairs daily functioning, with persistent nausea adversely affecting quality of life (Dean et al., 2018). Severe NVP is associated with anxiety, depression, suicidal ideation, and post-traumatic stress disorder (PTSD) (Kjeldgaard et al., 2019; Mitchell-Jones et al., 2017; Nana et al., 2022b; Nijsten et al., 2022b). Pre-existing mental health conditions may be exacerbated by NVP when oral medication cannot be taken or impacted by symptoms (Nelson-Piercy et al., 2024). Nutritional intake and ability to take micronutrient supplements have also been shown to be suboptimal in those affected, with intakes of energy, protein, fat, carbohydrate, fibre, folate, vitamin C, calcium, iron and zinc lower in women with severe sickness (Maslin et al., 2021). Furthermore, severe NVP has been reported to negatively affect relationships with partners and family (Beirne et al., 2023; Bulin, 2019; Heitmann et al., 2017; Mazzotta et al., 2001; O’Brien et al., 2002; Tian et al., 2017), while also contributing to absenteeism from work, financial pressures, and difficulty in caring for family (Beirne et al., 2023; Davis, 2004; Dørheim et al., 2013; Tian et al., 2017). Increased stress related to the perception of others that NVP is psychosomatic and reluctance and lack of confidence of some doctors to provide treatment has also been reported (Heitmann et al., 2016; Nana et al., 2022a, b).

Severe NVP can additionally affect short and long-term infant and child health. Observational data demonstrate that pregnancies of women with HG and low gestational weight gain are at an increased risk of preterm birth and low birthweight (Dodds et al., 2006; Jansen et al., 2023), while systematic review evidence reports a small increase in the risk of adverse outcomes, including anxiety, autism and attention deficit disorder in children born to women with HG (Nijsten et al., 2022).

Due to the significant burden of severe NVP and its high recurrence risk, tailored strategies to address the physical and mental health of those affected are needed during the preconception, pregnancy, and postpartum periods. Recent UK guidelines advise on the diagnosis and treatment of NVP, with recommendations to develop and assess interventions to improve mental health outcomes and evaluate pre-emptive intervention on severity and duration of symptoms in subsequent pregnancies (Nelson-Piercy et al., 2024). Preconception counselling has shown reductions in adverse pregnancy outcomes in women with chronic medical conditions (Nana et al., 2023), while the James Lind Alliance research priorities include pregnancy planning for women with previous HG (Dean et al., 2021). Furthermore, qualitative research has demonstrated the unmet need for care following a pregnancy with severe NVP and the need for a continuum of care from pregnancy to postpartum (Beirne et al., 2023). This study, therefore, aimed to conduct a scoping review to identify and characterise interventions that are initiated pre- and post-pregnancy that seek to mitigate the impact of severe pregnancy sickness on maternal health.

## Methods

This review followed the frameworks by Arksey and O’Malley (2005), Levac et al. (2010), and the Joanna Briggs Institute (JBI) (Peters et al., 2015). The Preferred Reporting Items for Systematic Reviews and Meta-Analysis extension for Scoping Reviews (PRISMA-ScR) guidance was used to report the review process (Supplementary information 1) (Tricco et al., 2018). The final protocol was registered prospectively in Open Science Framework (https://osf.io/ghcb7).

### Search Strategy

An extensive search of studies that investigated pre- and post-pregnancy interventions for women who experienced NVP was conducted in January 2024. A three-step search strategy was developed in collaboration with an information specialist (Supplementary information 2). In the first step, a preliminary search of Embase and Medline was conducted, where the text words in the titles and abstracts of retrieved articles, and the index terms used to describe the articles were analysed. A second search using all identified keywords and index terms was undertaken, without restriction to date or language, using the following databases: Embase, Medline, PsycINFO, CINAHL, Scopus, Web of Science, and the Cochrane Library. In addition to reference and citation searching, a third search focusing on grey literature was conducted through Trip pro, Mednar, Lenus The Irish Health Repository, electronic thesis and dissertation repositories (ProQuest platform, Bielefeld Academic Search Engine, The Networked Digital Library of Theses and Dissertations, and Open Access Thesis and Dissertations), pre-print servers (SSRN, PubMed preprints, MedRxiv, SSRN, and Europe PMC), as well as websites of international organisations such as Hyperemesis Education and Research (HER), Royal College of Obstetricians & Gynaecologists (RCOG), and the American College of Obstetricians and Gynaecologists (ACOG). The results of the search were exported to Endnote for deduplication and imported into Rayyan software (https://rayyan.qcri.org) for data management (Ouzzani et al., 2016).

### Eligibility Criteria

Studies that utilised an intervention, including but not limited to, lifestyle, psychological, or pharmacological, initiated in the pre- or post-pregnancy period for women who experienced severe NVP were included (Supplementary information 3). All types of study designs including randomised controlled trials, observational and qualitative studies, were deemed eligible. Systematic reviews and meta-analyses (except for the purpose of hand search), case reports, protocols, editorials, commentaries, conference proceedings, medical guidelines, and animal research were excluded.

### Data Collection and Analysis

Three reviewers (ZD, OB, KA) screened titles and abstracts of the articles against the eligibility criteria. Each article was assessed by at least two blinded reviewers, and any conflicts that arose were resolved through discussion with a fourth reviewer (ACF). Full texts were retrieved and screened, with reasons for exclusion reported. In cases where articles were not published in English, Google Translate was utilised for screening (Jackson et al., 2019).

The organisation of data from the included articles was carried out using Microsoft Excel. Data extraction was performed by one reviewer and validated by a second reviewer. Items extracted from each article included author, year of publication, study aim and design, target population, sample size, intervention content and delivery, and study findings. For qualitative study designs, content analysis was used to classify and summarise key characteristics of the intervention and its main outcomes (Pollock et al., 2023). Methodological appraisal of the literature and risk of bias assessments were not undertaken, consistent with JBI guidance, as the main aim of a scoping review is to describe and map the evidence rather than examine a specific research question (Peters et al., 2015).

## Results

**Fig. 1:**
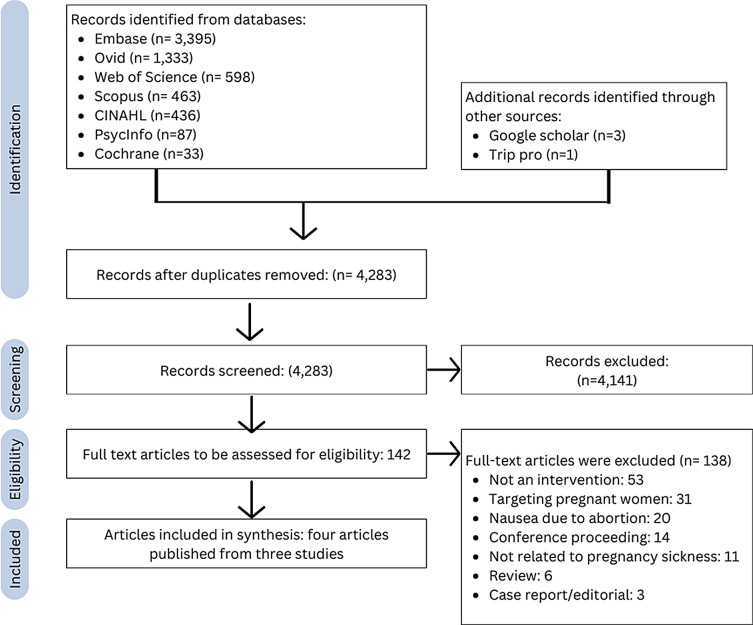
Study selection according to PRISMA-ScR guidelines^* *^ A study by Nicholson (2018, 2019) was reported across two articles

The results of the search yielded 6,349 articles, with 4,283 remaining following deduplication. Following title and abstract screening, the full texts of 142 articles were retrieved and screened (Fig. 1).

Three studies were included, which were reported across four articles. Two studies focused on pre-emptive counselling and pharmacological treatment initiated before and in early pregnancy (Koren & Maltepe, 2004; Maltepe & Koren, 2013), while one study utilised writing therapy after pregnancy (Nicholson, 2018, 2019) (Tables 1 and 2).

**Table 1:** Characteristics of the included studies

Study characteristics				Sample size		Age (years)*		Severity of NVP in previous pregnancy		Ethnicity/ Education
Author (Year)	Country	Study design	Sampling	Intervention	Control	Intervention	Control	Intervention	Control	
Koren and Maltepe (2004)	United States and Canada	Prospective matched trial	Convenience	25	35	32.3 (4.2)	31.9 (4.8)	HG (*n* = 18, 72%), severe NVP (*n* = 7, 28%)	HG (*n* = 5, 14.3%), severe NVP (*n* = 30)	Not reported
Maltepe and Koren (2013)	Not reported	RCT	Convenience	31 (one participant was excluded from the final sample due to insufficient data)	29	32.2 (4.7)	31.3 (3.2)	HG (*n* = 19, 63.3%), severe NVP (*n* = 11, 36.7%)	HG (*n* = 11, 37.9%), severe NVP (*n* = 18, 62.1%)	Not reported
Nicholson (2018, 2019)	United Kingdom	Not reported	Convenience	10	NA	32–48	NA	HG (*n* = 10, 100%)	NA	Not reported

NA: not applicable, * data presented as mean (SD) or range

**Table 2:** Description of interventions and outcomes

Author (Year)	Intervention type	Intervention description	Intervention outcomes
Koren and Maltepe (2004)	Pharmacological prophylactic treatment	The intervention group were counselled to commence antiemetic medication before conception or up to 7 weeks of gestation. In the control group, treatment with antiemetics began after NVP symptoms in 26 of the women, while 9 women did not take any antiemetic medication.	• No change in severity (i.e. severe– severe): pre-emptive group (*n* = 12, 48%), control group (*n* = 28, 80%), *p* = 0.01 • Improvement from (severe– moderate): pre-emptive group (*n* = 5, 20%), control group (*n* = 5, 14%) • Improvement (severe– mild): pre-emptive group (*n* = 8, 32%), control (*n* = 2, 13.8%), *p* < 0.05 • Reduction of HG recurrence: pre-emptive (from 18/25 before intervention to 8/25 after the intervention, *p* = 0.01), control (from 5/35 before the intervention to 3/35 after the intervention, not significant)
Maltepe and Koren (2013)	Pharmacological prophylactic treatment and counselling	The pre-emptive arm started with 2 tablets of doxylamine/pyridoxine at bedtime upon the recognition of pregnancy. The control arm started with 2 tablets of doxylamine/pyridoxine at bedtime on the first day of NVP symptoms. Both groups were advised to gradually increase their medication dosage if their symptoms escalated. Upon enrolment, both groups, including women planning pregnancy, received counselling on non-pharmacological symptom management, including optimising dietary and fluid intake, lifestyle advice, and advice on associated symptoms.	• Percentages of cases described as moderate-severe in the first 3 weeks: pre-emptive group (*n* = 4, 15.4%), control group (*n* = 9, 39.13%), *p* < 0.05 • NVP resolved before labour: pre-emptive group (78.2%), control group (50%), *p* < 0.002 • Gestational age when NVP resolved (median weeks): pre-emptive group (26), control group (33), *p* = 0.18 • Reduction of HG recurrence: pre-emptive group (from 19/30 to 6/30, 43.3%), control (11/29 to 6/29, 17.2%), *p* = 0.047
Nicholson (2018)	Writing therapy	Participants were invited to complete 4 separate 15-minute reflective writing tasks at home over a 2-week period. This was followed by a qualitative telephone interview focusing on their experiences of the writing process.	Four superordinate themes emerged from the data: writing as an opportunity to externalise the HG experience; writing as a space to process the HG experience; writing as a means of reclaiming relationship with self and others; writing as a place to heal and recover.
Nicholson (2019)		Same intervention study as above	Writing for the self, rather than for an audience, was found to be an adequate tool for making sense of one’s lived experience and reflecting on previously undisclosed topics. However, some participants reported challenges with this form of therapy, such as distress from revisiting vivid memories. Additionally, the absence of human connection in expressive writing was noted as a limitation, as it lacked the aspect of being heard and supported by others.

### Interventions Initiated in the Pre-Pregnancy Period

The first study, a prospective design, conducted by Koren and Maltepe between 2001 and 2003 in the USA and Canada, evaluated the effectiveness of pre-emptive treatment of NVP in reducing the rates of HG and severe NVP. HG was defined using the criteria set by Fairweather (1968) as a severe form of NVP that extends into the first 20 weeks of gestation plus hospitalisation for the treatment of dehydration, electrolyte imbalances, and vomiting. Participants were recruited through an NVP-Healthline to counsel women on the management of NVP symptoms. The intervention group (*n* = 25) included women with previous NVP who contacted the healthline for advice on preventing a similar experience in a future pregnancy. The comparison group (*n* = 35) consisted of pregnant women with previous NVP who contacted the healthline for advice on symptom management when symptoms began. Severity of NVP was assessed using the Pregnancy Unique Quantification of Emesis (PUQE) questionnaire. The intervention group were counselled to commence antiemetic medication before conception, or up to 7 weeks of gestation (for a range of 4-150 days). The medication regimen that the women received was heterogeneous and not standardised. In the control group, treatment with antiemetics began 1–63 days after NVP symptoms in 26 of the women, while 9 women did not take any medicinal antiemetic medication. In the intervention group, there was a significant improvement in NVP severity compared to the previous pregnancy (*p* < 0.05), and a reduction in HG (8 vs. 18, *p* < 0.05).

The second study, by the same authors in 2013, was a randomised controlled trial to compare the effectiveness of the pre-emptive use of 10 mg doxylamine/10 mg pyridoxine in women who had experienced severe NVP in their previous pregnancy, to women with a similar previous experience, receiving antiemetic medication only on the first sign of nausea. HG was defined as severe NVP that requires admission to the emergency room or hospitalisation for severe symptoms. The study utilised pre-emptive lifestyle counselling and pharmacological treatment. The study recruited women from the NVP-Healthline. Some women were not pregnant but planning pregnancy, while others were pregnant at the time of recruitment. Upon enrolment, all women, including women planning pregnancy, received specific counselling, which focused on optimising dietary and fluid intake, lifestyle advice, e.g. minimising nauseating stimuli and advice on associated symptoms, e.g. managing gastrointestinal symptoms and supplemental intake. Women participated in weekly and as needed telephone follow-up counselling and were reminded to contact the study coordinator as soon as they conceived and when symptoms began. They were randomised in a 1:1 ratio to receive antiemetic medication when pregnancy was confirmed and before NVP symptoms began (*n* = 31) or to begin antiemetic medication treatment on the first day of NVP symptoms (*n* = 29). Severity of NVP symptoms was assessed using PUQE scores. The primary endpoint was the change in rates of HG in the index pregnancy compared to previous pregnancies within each group. Pre-emptive use of antiemetic medication reduced recurrence of HG by 43.3% between previous (19/30) and the index pregnancy (6/30), compared to 17.2% in the control group (from 11/29 to 6/29, *p* < 0.05). Symptom severity was also reduced in the intervention arm; there were 70% fewer cases of moderate-severe NVP in comparison to the control group (39.13%) during the 3 first weeks of NVP (*p <* 0.05), in addition to more women in whom NVP resolved before birth compared to the control group (*p* < 0.05).

### Interventions Initiated in the Post-Pregnancy Period

One post-pregnancy study, conducted in the UK, evaluated whether expressive writing, a form of writing therapy, might be helpful for women who experienced NVP (Nicholson, 2018). Participants were recruited through a pregnancy sickness support charity, and inclusion criteria were women aged 18 or above who had previous experience of any degree of pregnancy sickness. Ten participants were recruited, aged between 32 and 48 years, and their most recent HG experience ranged from 1 to 9 years prior to the study. All 10 women had been hospitalised at least once with severe NVP. The women were asked to write reflectively about their pregnancy sickness at home on four occasions for two weeks. Participants were provided with written guidelines and instructed to write reflectively about their experiences, thoughts, and feelings. Qualitative data were collected using semi-structured telephone interviews, and data was analysed using thematic analysis. The study reported a positive effect of expressive writing as a self-help tool for the participants to reclaim the narrative around their experiences. Four themes were identified, which centred around writing as an opportunity to externalise the HG experience, writing as a space to process the HG experience, writing as a means to reclaim relationships, and writing as a way to heal. The participants spoke about how writing allowed them to express feelings and thoughts not previously disclosed: ‘what I’ve not been able to articulate’, while allowing them to make sense of the experience: ‘the more I’ve written, the more I’ve been able to say, wow, it’s no wonder you felt like that’. For some participants, writing increased sense of agency: ‘When you’re that ill, you feel totally out of control of your own body...when you write it down, it kind of makes you feel a bit more in control’, while for other participants, writing was a way to recover: ‘It’s like a physical release.. almost like a pain barrier to go through’. In the second article for the same study (Nicholson, 2019), benefits and limitations of expressive writing were further explored. Although expressive writing helped women, it lacked the relational aspect offered by a therapist or conversations with other women who had similar experiences, leaving some women feeling unheard and invalidated, despite their desire for this connection. For some women, remembering the experience was stressful: ‘it [was] quite hard to be sad again and be in touch with those feelings again.’(Nicholson, 2019).

## Discussion

This scoping review aimed to identify and characterise interventions initiated before and after pregnancy for women with previous severe NVP. The findings identified three studies: pre-emptive counselling and antiemetic treatment beginning before pregnancy and in early pregnancy/upon recognition of pregnancy and one post-pregnancy writing therapy intervention. The pre-emptive interventions reported a reduction in NVP symptom severity and a lower recurrence rate of HG in women who took pre-emptive antiemetic medication in a subsequent pregnancy. Writing therapy was beneficial in aiding recovery from severe NVP, and allowed women an opportunity to externalise and process the experience. The limited number of studies highlights the paucity of interventions available for women who have had pregnancies complicated by severe NVP.

The recurrence rate of HG ranges from 15 to 81% (Dean et al., 2020). While many women with previous severe NVP report not wanting to have a future pregnancy (Havnen et al., 2019), it is critical that pregnancy preparation and preconception health are optimised for those planning future pregnancies. The current review identified two studies, conducted by the same authors, that utilised pre-emptive antiemetics and tailored counselling, which began before pregnancy for some participating women. The findings showed that early use of antiemetics and counselling on dietary and lifestyle modifications resulted in a lower recurrence of HG and less severe symptoms (Koren & Maltepe, 2004; Maltepe & Koren, 2013). However, both studies had limitations, including small sample sizes. A recent review highlights the safety of medication used to treat NVP, including the combination of doxylamine/pyridoxine used in the 2013 study by Maltepe & Koren (Nelson-Piercy et al., 2024). As preconception counselling has beneficial effects on pregnancy outcomes in women with chronic conditions (Nana et al., 2023), further research is needed on a personalised approach to pre-pregnancy planning and care for women with previous severe NVP.

For many women, physical symptoms of NVP can start before a missed period and potentially last for the duration of pregnancy. Together with the impact of the condition on psychosocial factors, such as the inability to care for family, impact on relationships, finances and employment (Beirne et al., 2023; Davis, 2004; Dørheim et al., 2013; Tian et al., 2017) and pregnancy termination (Nana et al., 2021; Nurmi et al., 2019), pre-pregnancy preparation and optimisation are critical for women with previous severe NVP. Furthermore, the importance of the preconception period is well recognised for improving maternal health behaviours, and reducing complications in pregnancy (Nana et al., 2023). Preconception health behaviours have been demonstrated to be suboptimal in the general population of women (Hanson et al., 2015; Lang et al., 2021; Stephenson et al., 2018), particularly suboptimal intakes of key pregnancy micronutrients including folate, vitamin D, calcium, iron and iodine (Stephenson et al., 2018). Strategies to optimise pregnancy preparation in women with previous NVP is particularly important in those vulnerable to inadequate nutrition in pregnancy, such as women with severe NVP.

Women describe severe NVP as one of their worst life experiences (Havnen et al., 2019). Yet, despite the physical and mental health consequences of NVP (Attard et al., 2002; Beirne et al., 2023; Bulin, 2019; Heitmann et al., 2017; Kramer et al., 2013; Maslin & Dean, 2022; Nijsten et al., 2022b; O’Brien et al., 2002), only one post-pregnancy intervention was identified in this review. The intervention used writing therapy to address emotions and issues around former pregnancy sickness, and was evaluated using a qualitative study design (Nicholson, 2018, 2019). Writing therapy is considered a standalone intervention or can be used in addition to other therapies (Ruini & Mortara, 2022). It has been shown to be effective in reducing distress and traumatic symptoms in the postpartum population (Lim et al., 2024; Liu et al., 2014; Qian et al., 2020). The benefits of writing therapy were described by the participants in Nicholson, and included providing an opportunity to externalise their experience, to process the experience, to reclaim relationships, and to help with recovery. The findings indicate that emotional recovery is an ongoing process for women after pregnancy, even when the physical symptoms of severe NVP have dissipated. Further research is needed to address mental health following NVP, particularly as mental ill health can persist postpartum and HG is a risk factor for postpartum PTSD (Christodoulou-Smith et al., 2011; Kjeldgaard et al., 2019; Nijsten et al., 2022b).

No studies which targeted physical health after severe NVP were identified in this review. There is a lack of data on postpartum physical health following a pregnancy complicated by severe NVP, yet, nutrient deficiencies have been reported during pregnancy, even in those with mild symptoms (Maslin et al., 2021). The effect of severe NVP on postpartum nutritional status is not clear, and how nutritional status impacts maternal and infant health after pregnancy (Maslin et al., 2021; Santander Ballestín et al., 2021). Inability to eat or drink normally can lead to feelings of guilt in women with NVP, who are concerned that sub-optimal nutritional intake during pregnancy may affect fetal development and impact breastfeeding (Beirne et al., 2023). Women who experience severe NVP are more likely to report dental issues, and menstrual cycle irregularities (Tian et al., 2017), in addition to persistent muscle aches, digestive issues, and continued nausea and vomiting in the postpartum period (Bulin, 2019). Together, this indicates the need for further research to understand how severe NVP impacts physical health postpartum in order to develop strategies to address physical health optimisation post pregnancy.

## Strengths and Limitations

This review has several strengths. A comprehensive search strategy was developed with assistance from an information specialist. All article screening and data extraction stages were conducted in duplicate by independent reviewers. Limitations of the review include the potential for publication bias, which may have occurred as some studies might be published through conference abstracts or within organisational reports and might not have been captured in this review. The interpretation was impacted by limited data availability from two jurisdictions, which might not reflect practices globally. In addition, the inclusion of mixed cohorts of women, some who were not pregnant but planning pregnancy and those already pregnant in the active arms, impacted interpretation of interventions initiated in the pre-pregnancy period. Finally, although critical appraisal is not needed for a scoping review, it limits confidence in the studies included.

## Conclusion

Due to the significant burden of severe NVP, and the high recurrence risk in subsequent pregnancies, there is an urgent need for evidence-based interventions that optimise physical and mental health before and after pregnancy. This study revealed a paucity of interventions initiated before and after pregnancy for women with previous severe NVP. The included interventions showed some benefits of pre-emptive counselling and pharmacological treatment on symptom severity and HG recurrence rates while writing therapy aided recovery. Tailored pre-pregnancy, and postpartum interventions for women with previous severe NVP are urgently needed to address the physical and mental health burden of the condition.
